# Novel Methods and Data Sources for Surveillance of State-Level Diabetes and Prediabetes Prevalence

**DOI:** 10.5888/pcd14.160572

**Published:** 2017-11-02

**Authors:** Russ Mardon, David Marker, Jennifer Nooney, Joanne Campione, Frank Jenkins, Maurice Johnson, Lori Merrill, Deborah B. Rolka, Sharon Saydah, Linda S. Geiss, Xuanping Zhang, Sundar Shrestha

**Affiliations:** 1 Westat, Inc, Rockville, Maryland; 2 Centers for Disease Control and Prevention, Atlanta, Georgia

## Abstract

States bear substantial responsibility for addressing the rising rates of diabetes and prediabetes in the United States. However, accurate state-level estimates of diabetes and prediabetes prevalence that include undiagnosed cases have been impossible to produce with traditional sources of state-level data. Various new and nontraditional sources for estimating state-level prevalence are now available. These include surveys with expanded samples that can support state-level estimation in some states and administrative and clinical data from insurance claims and electronic health records. These sources pose methodologic challenges because they typically cover partial, sometimes nonrandom subpopulations; they do not always use the same measurements for all individuals; and they use different and limited sets of variables for case finding and adjustment. We present an approach for adjusting new and nontraditional data sources for diabetes surveillance that addresses these limitations, and we present the results of our proposed approach for 2 states (Alabama and California) as a proof of concept. The method reweights surveys and other data sources with population undercoverage to make them more representative of state populations, and it adjusts for nonrandom use of laboratory testing in clinically generated data sets. These enhanced diabetes and prediabetes prevalence estimates can be used to better understand the total burden of diabetes and prediabetes at the state level and to guide policies and programs designed to prevent and control these chronic diseases.

## Introduction

Accurate estimates of diabetes and prediabetes prevalence are essential for monitoring the impact of these conditions on population health and for assessing the effectiveness of prevention programs (1). Diabetes was the seventh leading cause of death in 2014; it can lead to severe complications, and, in 2012, it cost approximately $176 billion nationally in medical care (2). In 2012, among US adults aged 20 years or older, the prevalence of diabetes was 12.3% and the prevalence of prediabetes was 37% (3). Many public health efforts for diabetes prevention, education, and risk-factor control occur at the local and state level, and prevalence estimates vary widely by geography (4). However, state-level estimates of diabetes and prediabetes prevalence that include undiagnosed cases are often unavailable because of a lack of applicable data. In this article, we describe testing the feasibility of using existing data sources in novel ways to enhance surveillance of diabetes and prediabetes prevalence at the state level.

The most accurate and useful diabetes surveillance methods represent a population, identify both diagnosed and undiagnosed cases (via biomarkers such as fasting plasma glucose [FPG] or hemoglobin A1c [HbA1c]), and include geographic, demographic, and risk variables to support adjustment and subpopulation analysis. Because of their high cost, such methods are typically conducted at the national level. An example is the National Health and Nutrition Examination Survey (NHANES) (5,6). NHANES, because of the small sample size in each state, is not designed to assess state-level prevalence directly (7). At the state level, the most commonly used surveillance tool for assessing chronic disease prevalence is the Behavioral Risk Factor Surveillance System (BRFSS), an annual telephone survey (8). However, BRFSS is unable to identify cases of undiagnosed diabetes, and it underreports prediabetes since many respondents are unaware that they have the condition (9).

In recent years, the availability and potential use of other types of data has increased. Several surveys that include FPG or HbA1c measurements, or both, have large sample sizes that can support state-level surveillance in some states. These data sources include the Health and Retirement Study (HRS) and the National Ambulatory Medical Care Survey (NAMCS). Furthermore, the increasing availability of large administrative data sets produced during routine health care delivery and the continual growth and standardization of electronic health records make these 2 data sources potentially useful for disease surveillance (9–19).

Although these data sources have great potential for state-level surveillance, they raise methodologic challenges. First, they often cover nonrandom subpopulations that are not always clearly specified. Second, the services or tests conducted may be determined by individual health care needs rather than by any research design; thus, the data may have many nonrandom gaps. Third, administrative and clinical data often have inconsistencies in coding across providers, and claims data may have biases caused by their use in billing (20,21). Fourth, differences in specimen collection or laboratory methods can affect results (22). Fifth, any service-based data set does not have information on people who do not have contact with the health care system. Finally, these data sets often lack key demographic variables, such as race and income, that are associated with diabetes and are useful for modeling and adjustment. In this article, we introduce an approach to reducing some of these biases. We illustrate our methods by applying them to data from 2 states, California and Alabama, which vary in size, demographic characteristics, diabetes prevalence, and data richness. In 2014, according to BRFSS data, the crude prevalence of diagnosed diabetes among adults was 10.3% in California and 12.9% in Alabama (8).

## Data Sources

All analyses for this research took place from July 2015 through March 2017. We assessed a range of administrative, clinical, and survey data sources, and for each data set, we summarized information on the adjustments needed to improve representativeness of the state-level population, characterized the type of data included, described how the surveillance data are used, and listed the covariates included (Table 1).

**Table 1:** Assessment of Data Sets for Surveillance of Diabetes and Prediabetes in the United States^a^

Assessment	NHANES 2011–2012 (Public-Use File)	HRS 2012 (Confidential Data Request)	NAMCS 2012 (Public-Use File)	MarketScan 2013 (Proprietary Data Product)
**Adjustments needed to improve representativeness of state-level population**				
State-level	No	Some states	Some states	Some states
Population-based	Yes	Yes for age >50 y	Yes, for those with office visits	No, observational
Undiagnosed cases	Yes, HbA1c and fasting plasma glucose	Yes, HbA1c only	Yes, but a nonrandom laboratory subset	Yes, but a nonrandom laboratory subset
Covariates	Yes	Yes	Limited	Limited
**Types of data included in each data set**				
Patient survey	Yes	Yes	No	No
Administrative, including inpatient and outpatient claims data	No	No	No	Yes
Clinical, including electronic medical records and patient chart reviews	No	No	Yes	No
Includes medications	Yes	No	Yes	Yes
Includes laboratory values for HbA1c and/or fasting plasma glucose tests	Yes	Yes	Yes	Yes
**How the data set is used for surveillance**				
Self-reported diabetes	Yes	Yes	No	No
Clinician-diagnosed diabetes	No	No	Yes	Yes
Undiagnosed diabetes	Yes	Yes	Yes	Yes
Self-reported prediabetes	Yes	No	No	No
Clinician-diagnosed prediabetes	No	No	No	Yes
Undiagnosed prediabetes	Yes	Yes	Yes	Yes
**Covariates included in the data set**				
Age	Yes	Yes	Yes	Yes
Sex	Yes	Yes	Yes	Yes
Race/ethnicity	Yes	Yes	Yes	No
Education	Yes	Yes	No	No
Income	Yes	Yes	No	No
Insurance type	Yes	Yes	Yes	Yes

Abbreviations: HbA1c, hemoglobin A1c; HRS, Health and Retirement Study; NAMCS, National Ambulatory Medical Care Survey; NHANES, National Health and Nutrition Examination Survey.

a Assessment conducted from July 2015 through March 2017.

NHANES (https://www.cdc.gov/nchs/nhanes/) is a publicly available population-based survey data set that includes the biomarkers needed to identify undiagnosed cases. It has many covariates available for adjustment, but it is a national data set rather than a state-level data set.

HRS (http://hrsonline.isr.umich.edu/) is a longitudinal panel study representing Americans aged over 50 years. In addition to data on self-reported diabetes, HRS collects data on HbA1c tests, which can identify undiagnosed cases (18,23,24). Although HRS is not designed to be representative of any individual state, it can be used for direct state-level estimation for some states where the sample size is sufficient, and the survey includes many covariates for adjustment. HRS is available at no cost; however, one must apply for access to state identifiers, and the security requirements for use of this variable are stringent.

NAMCS (http://www.cdc.gov/nchs/ahcd/) is a nationally representative survey of physician office visits. It contains data on risk factors (age, sex, race, and ethnicity), laboratory results, ambulatory health care services, medications, diagnoses, and a list of diseases, including diabetes, as recorded in the physician office’s patient medical record. The use of laboratory testing is nonrandom, determined by routine clinical decision making. In 2012, the sample size of NAMCS was increased to allow for state-level estimation in the most populous states. NAMCS data are publicly available, but they have a smaller set of covariates for making adjustments than NHANES or HRS have.

MarketScan, licensed by Truven Health Analytics, is a large proprietary database of commercial inpatient, outpatient, and pharmacy claims from approximately 100 payers representing large employers and health plans. For some patients, MarketScan has data on laboratory results useful for identifying undiagnosed cases of diabetes or prediabetes. Like HRS and NAMCS, MarketScan can be used for direct state-level estimation in states where the number of observations is sufficient. It shares with NAMCS the limitations of nonrandom data on laboratory testing, and it has an even smaller set of covariates than NAMCS. MarketScan has the additional complexity of being an observational data set, whereby inclusion is voluntary and not determined by a population sample design. The cost of acquiring MarketScan data varies, depending on the state and the files needed.

## Approaches for Reducing Bias to Improve State-level Population Representativeness

We harmonized variables across the 4 data sets so that similar concepts were coded consistently by using SAS version 9.1.3 (SAS Institute, Inc) for all analyses. We repeatedly relied on 2 basic adjustment techniques: propensity adjustment and raking (25,26). These methods are used to reweight observations in a focal data set (eg, NHANES, HRS, NAMCS, MarketScan) to increase the data set’s representativeness of a target population (eg, a state’s population). Raking can achieve a highly exact match on the distributions of covariates when a few covariates are used. Propensity adjustment can accommodate a larger number of covariates than raking can, and it can accommodate interactions among covariates (27–29).

Diagnosed cases of diabetes and prediabetes were based on survey self-report, selected ICD-9-CM (*International Classification of Diseases, 9th Revision, Clinical Modification*) diagnosis codes (30), and selected diabetes medications (Table 2). Undiagnosed cases of diabetes were based on an HbA1c value of 6.5% or greater or an FPG value of 126 mg/dL or greater, while undiagnosed cases of prediabetes were based on an HbA1c value ranging from 5.7% to less than 6.5% and an FPG value ranging from 100 mg/dL to less than 126 mg/dL.

**Table 2:** ICD-9-CM Diagnosis Codes and Medications Indicating Diabetes or Prediabetes^a^

Code or Medication	Description
**Codes for diabetes**	
250.x0	Diabetes mellitus without mention of complication, type II or unspecified type, not stated as uncontrolled (250.00) Diabetes with ketoacidosis, type II or unspecified type, not stated as uncontrolled (250.10) Diabetes type with hyperosmolarity, type II or unspecified type, not stated as uncontrolled (250.20)
250.x1	Diabetes mellitus without mention of complication, type I [juvenile type], not stated as uncontrolled (250.11) Diabetes with renal manifestations, type I [juvenile type], not stated as uncontrolled (250.41)
250.x2	Diabetes mellitus without mention of complication, type II or unspecified type, uncontrolled (250.02) Diabetes with ketoacidosis, type II or unspecified type, uncontrolled (250.12) Diabetes with peripheral circulatory disorders, type II or unspecified type, uncontrolled (250.72)
250.x3	Diabetes with neurological manifestations, type I [juvenile type], uncontrolled (250.63)
357.2	Polyneuropathy in diabetes
362.0x	Diabetic retinopathy
366.41	Diabetic cataract
648.0x	Diabetes mellitus of mother, complicating pregnancy, childbirth, or the puerperium, unspecified as to episode of care (not gestational diabetes)
**Code for prediabetes**	
790.29	Abnormal glucose not elsewhere classified
**Medications**	
Alpha-glucosidase inhibitors	
Amylin analogs	
Insulin among nonpregnant women	
Antidiabetic agent combinations including those with metformin	
Meglitinides	
Sodium glucose cotransporter 2 (SGLT2) inhibitors	
Sulfonylureas or thiazolidinediones	

Abbreviation: ICD-9-CM, *International Classification of Diseases, 9th revision, Clinical Modification* (30).

a Assessment conducted from July 2015 through March 2017.

### Adjustments to NHANES data

**Step 1: Demographic adjustment.** To adjust NHANES national weights to reflect the demographic characteristics of a state, we first raked BRFSS to the American Community Survey (ACS) on age, sex, race/ethnicity, insurance type, education, marital status, and income. We then used propensity modeling to adjust NHANES to the ACS-adjusted BRFSS on these same variables, plus self-reported answers to the general health question (response options for which ranged from excellent to poor). This 2-step process creates state-level NHANES weights that account for possible differences in health status across states within demographic subgroups.

**Step 2: Imputation of FPG range.** Half of the NHANES sample includes both HbA1c and FPG measurements, while the other half of the sample includes only HbA1c measurements. Having only HbA1c measurements introduces the possibility of a downward bias in prevalence estimates, because there is only one way to detect undiagnosed diabetes. We used the half of the sample with both measurements to fit 2 logistic regression models, one to predict whether FPG indicates diabetes (≥126 mg/dL) and one to predict whether FPG indicates prediabetes (100 mg/dL to <126 mg/dL). We found the models fit well (95% concordance [*C* = .946]) after taking the logarithms of both blood measurements, using as predictors HbA1c, HbA1c-squared, body mass index, sex, age, age-squared, insurance type, race/ethnicity, income, and general health and all 2-way interactions of these variables. We then applied the fitted models to the remaining half of the NHANES sample to impute the FPG range for those for whom it was not measured.

**Step 3: Case definition.** We defined cases according to self-reported answers to the questions about diagnosed diabetes and diagnosed prediabetes (ever been told have diabetes or prediabetes) and the HbA1c and FPG measurements (FPG range imputed where missing) for undiagnosed cases.

### Adjustments to HRS data

**Step 1: Selection of respondents.** Some states, such as California, have a sufficient number of respondents to base estimates solely on respondents from that state. Alabama does not have a sufficient number of respondents, so we used data from respondents from all 4 states in the East South Central census division (Alabama, Kentucky, Mississippi, and Tennessee).

**Step 2: Demographic adjustment. **We followed the same process for HRS as we did for NHANES in Step 1: we used HRS weights as the starting point and a target population aged over 50 years.

**Step 3: Imputation of FPG range. **HRS collects data on HbA1c measurements but not FPG measurements. Again, having only HbA1c measurements introduces the possibility of a downward bias in prevalence estimates. Because we harmonized the variable coding across data sets, we could apply the NHANES imputation equations (NHANES Step 2) to HRS. This process yielded a predicted FPG range for each HRS respondent.

**Step 4: Case definition.** We defined cases according to self-reported answers to the questions about diagnosed diabetes (ever been told have diabetes) and HbA1c and FPG measurements (imputed) for undiagnosed diabetes. HRS does not have a question on self-reported prediabetes, so prediabetes was identified solely by examining laboratory values.

### Adjustments to NAMCS data

**Step 1: Person-level weight adjustment.** The weights provided in NAMCS are designed for analysis at the level of the office visit, so people who visit a physician office more often are overrepresented. We adjusted weights so that each person was equally weighted, by dividing the office-visit weight by the number of office visits per person (31).

**Step 2. Selection of respondents.** As we did in Step 1 for HRS, we determined whether we could make estimates solely on the basis of data from NAMCS respondents in that state (California) or if we had to use data from respondents in the surrounding states in the same census division (Alabama).

**Step 3: Demographic adjustment.** We followed the same process described in Step 1 for NHANES, using the NAMCS weights as the starting point; however, the target population was people who had an office visit in the previous 12 months, a subset that can be identified from BRFSS.

**Step 4. Case definition for diagnosed diabetes.** NAMCS has a unique variable for diagnosed diabetes: the variable is based on clinician review of all of the information available in the medical record. NAMCS also includes up to 3 diagnosis codes as well as prescription drug information to identify diagnosed diabetes.

**Step 5: Case definition for undiagnosed diabetes and prediabetes.** NAMCS is more complex than NHANES or HRS in that the use of laboratory testing is determined by clinical decision making. Depending on the state, approximately 15% to 20% of patients have at least 1 diabetes laboratory test (HbA1c or FPG), and this is not a random subset. We used propensity adjustment to account for this bias by adjusting for covariates associated with the occurrence of testing among people not diagnosed with diabetes or prediabetes. The variables age, body mass index, number of physician visits, hospitalization, disability, primary care physician, private insurance, race/ethnicity, practice ownership, and number of medications had a significant association with the use of laboratory tests for diabetes. We then reweighted the nondiagnosed subpopulation so that the weights for the subgroup that received diabetes laboratory testing aligned with the full nondiagnosed subpopulation. The weights for people who were not diagnosed and did not receive diabetes laboratory tests were set to zero.

### Adjustments to MarketScan data

MarketScan includes several linked files, including enrollment information (age, sex, and insurance benefits), claims information (diagnosis, procedure, and pharmacy codes for services delivered), and a laboratory file (HbA1c and FPG test results). In states where MarketScan data are available, a sufficient number of records typically exists to make estimates based on records from that state alone.

**Step 1: Demographic adjustment. **The first step was to adjust the enrollment file to state demographics as in Step 1 for NHANES. MarketScan has national weights derived from the household component of the Medical Expenditure Panel Survey (MEPS) that reflect the number of people who have employer-sponsored private health insurance. We adjusted these weights directly to the state ACS data according to age, sex, and metropolitan statistical area, because MarketScan does not include any other demographic variables or a general health variable. We chose as the target the ACS subpopulation that was employed, commercially insured, and aged 18 to 64, and earned at least $25,000 per year, a proxy for a job that would likely include insurance benefits.

**Step 2: Case definition for diagnosed diabetes and prediabetes**. We defined cases according to the ICD-9-CM diagnosis codes and medications indicating diabetes or prediabetes (Table 2).

**Step 3: Case definition for undiagnosed diabetes and prediabetes.** Because the availability of diabetes laboratory test results is determined by clinical decision making, we used a modeling process similar to the process described in Step 5 for NAMCS to estimate the prevalence of undiagnosed diabetes and prediabetes. The only variables available for the development of the model were age, sex, and urban location.

## Prevalence Estimates for Diabetes and Prediabetes in Alabama and California 

Our adjustments to NHANES data made a noticeable difference between Alabama and California in the prevalence rates of diagnosed diabetes; the prevalence was 4.1 percentage points higher in Alabama (11.8%) than in California (7.7%) (Table 3). In the adjusted HRS data set, the overall prevalence of diagnosed diabetes was much higher than in the NHANES population (25.3% in Alabama and 22.8% in California), whereas the difference between the 2 states in HRS (2.5 percentage points) was roughly consistent with the difference between the 2 states in NHANES. The prevalence of diagnosed diabetes in the NAMCS population (13.3% in Alabama and 10.0% in California) was higher than the prevalence in the NHANES population in both states. The prevalence of prediabetes (diagnosed and undiagnosed) in the NAMCS data was lower than in NHANES in both states (NAMCS, 28.5% in Alabama and 26.2% in California; NHANES, 40.7% in Alabama and 40.1% in California). In California, the prevalence of diagnosed and undiagnosed diabetes (7.8%) and diagnosed and undiagnosed prediabetes (18.5%) was lower in the MarketScan population than in the other data sets. MarketScan data were not available for Alabama.

**Table 3:** Prevalence of Diabetes and Prediabetes in Two States, Alabama and California,^a^ in Test of Feasibility of Using These Databases in Novel Ways to Improve Surveillance of Diabetes and Prediabetes^b^

Database/State	Diabetes, %			Prediabetes, %		
	Diagnosed	Undiagnosed	Both Diagnosed and Undiagnosed	Diagnosed	Undiagnosed	Both Diagnosed and Undiagnosed
**National Health and Nutrition Examination Survey 2011–2012 (full US population)**						
California	7.7	3.3	11.0	4.2	35.9	40.1
Alabama	11.8	2.6	14.4	5.2	35.5	40.7
**Health and Retirement Survey 2012 (US population aged >50 y)**						
California	22.8	3.5	26.3	NA	NA	37.5
Alabama	25.3	4.0	29.3	NA	NA	31.9
**National Ambulatory Medical Care Survey 2012 (had an office visit)**						
California	10.0	4.1	14.1	NA	NA	26.2
Alabama	13.3	10.5	23.8	NA	NA	28.5
**MarketScan 2013 (commercially insured US population aged 18–64 y)**						
California	5.5	2.3	7.8	0.3	18.2	18.5
Alabama	NA	NA	NA	NA	NA	NA

Abbreviation: NA, not available.

a These 2 states were chosen for assessment because they vary in size, demographic characteristics, diabetes prevalence, and data richness.

b Assessment conducted from July 2015 through March 2017.

## Discussion

This analysis demonstrated a feasible and novel approach for making use of nontraditional data sources for estimating the state-level prevalence of diabetes and prediabetes. We adjusted the data from 4 data sets designed for other purposes (other than the purpose of estimating state-level diabetes or prediabetes prevalence) to reduce several types of bias, thereby making the data sets more representative of state populations than unadjusted data sets are. These methods can be adapted and applied to a range of other survey, administrative, or clinical data sets that contain the diabetes laboratory values needed for surveillance of diagnosed and undiagnosed diabetes and prediabetes at the state or local level. Each data source has characteristic strengths and limitations.

NHANES is based on a national population sample, includes diabetes laboratory values for all members of the sample, has many covariates useful for analysis and adjustment, and is publicly available. The methods used in our feasibility test can be used to adjust NHANES data to better represent any state population. Furthermore, NHANES is the only data set we examined that includes individuals of all ages who are not in regular contact with the health care system. Our results for California and Alabama indicate that our adjustments to NHANES data made a noticeable difference in prevalence rates between the 2 states. However, the adjustments cannot capture unmeasured state-specific factors that may be associated with diabetes risk or prevalence. For example, if a state has a particularly effective diabetes prevention program, its impact will not be captured by national survey data. Similarly, if a racial or ethnic subgroup (eg, Hispanic people) in a state has higher diabetes risk than the nation because of differences within the subgroup, this difference will not be captured.

HRS has the advantage of supporting direct state estimation for some states for the population aged over 50, addressing this limitation of NHANES. The higher overall prevalence of diagnosed diabetes in the HRS population compared with the NHANES population was expected because the HRS population is an older population. The adjustments to HRS data are somewhat more complex than for NHANES, and HRS does not offer data on self-reported prediabetes.

NAMCS can support direct state estimation for some states for the population that has contact with the ambulatory health care system. It has a uniquely strong variable for diagnosed diabetes, and it is publicly available. However, it is affected by bias caused by the nonrandom use of diabetes laboratory tests, a complexity shared by other administrative or clinical data sets; this nonrandom use of laboratory tests necessitates several additional modeling steps and may not remove all bias. The prevalence of prediabetes was lower in the NAMCS population than in the NHANES population probably because NAMCS lacks an indicator for diagnosed prediabetes.

MarketScan can also support direct state estimation for states where there is sufficient participation. Unlike NHANES, HRS, and NAMCS, MarketScan is not sample-based, but because employer and laboratory participation is voluntary and anonymous, it is challenging to understand exactly what population is represented in the data. We made the assumption that the data represented those who were employed, commercially insured, aged 18 to 64 years, earned at least $25,000 per year, and had contact with the health care system. However, we could not assess the representativeness of the data in terms of employer industry, company size, or employee geographic location within state. As NAMCS does, MarketScan uses nonrandom data on diabetes laboratory testing, with the additional potential bias that not all clinical laboratories report results in the data set. Because MarketScan has fewer variables available for modeling, the adjustments for determining rates of undiagnosed diabetes and prediabetes are likely less robust than they are for the other data sets examined in this study. 

Although offering a feasible and flexible approach for adjusting data sets to reduce bias for diabetes prevalence estimation at the state level, the methods described in this article require considerable resources for data acquisition and analyses and may not completely account for the inherent biases and coverage limitations of the data sources. Our prevalence estimates have general face validity, but we cannot validate these estimates directly because of the absence of a gold standard. We are testing and validating a method to combine the state-level estimates of diabetes and prediabetes prevalence across these data sets to create a single composite prevalence estimate for a state. That analysis will allow us to assess the ability of each data source to contribute information that reduces bias and improves the precision of prevalence estimates at the state level, and it will help inform decisions about the optimal use of these data sources for state-level diabetes surveillance.
